# The efficiency of chlorophyll fluorescence as a selection criterion for salinity and climate aridity tolerance in barley genotypes

**DOI:** 10.3389/fpls.2024.1324388

**Published:** 2024-05-28

**Authors:** Zied Hammami, Soumaya Tounsi-Hammami, Nhamo Nhamo, Saleh Rezgui, Yousef Trifa

**Affiliations:** ^1^ Crop Diversification and Genetics Section, International Center for Biosaline Agriculture, Dubai, United Arab Emirates; ^2^ Department of Life and Environmental Sciences, College of Natural and Health Sciences at Zayed University, Dubai, United Arab Emirates; ^3^ Department of Agronomy and Biotechnology, Carthage University, National Agronomic Institute of Tunisia (INAT), Tunis, Tunisia; ^4^ Laboratory of Genetics and Cereal Breeding, National Agronomic Institute of Tunisia, Carthage University, Tunis, Tunisia

**Keywords:** salt tolerance, heat stress, barley, photosynthetic, North Africa

## Abstract

**Introduction:**

In the Near East and North Africa (NENA) region, crop production is being affected by various abiotic factors, including freshwater scarcity, climate, and soil salinity. As a result, farmers in this region are in search of salt-tolerant crops that can thrive in these harsh environments, using poor-quality groundwater. The main staple food crop for most of the countries in this region, Tunisia included, is barley.

**Methods:**

The present study was designed to investigate the sensitivity and tolerance of six distinct barley genotypes to aridity and salinity stresses in five different natural field environments by measuring their photosynthetic activity.

**Results and discussion:**

The results revealed that tolerant genotypes were significantly less affected by these stress factors than sensitive genotypes. The genotypes that were more susceptible to salinity and aridity stress exhibited a significant decline in their photosynthetic activity. Additionally, the fluorescence yields in growth phases J, I, and P declined significantly in the order of humid environment (BEJ), semi-arid site (KAI), and arid environment (MED) and became more significant when salt stress was added through the use of saline water for irrigation. The stress adversely affected the quantum yield of primary photochemistry (φP0), the quantum yield of electron transport (φE0), and the efficiency by trapped excitation (ψ0) in the vulnerable barley genotypes. Moreover, the performance index (PI) of the photosystem II (PSII) was found to be the most distinguishing parameter among the genotypes tested. The PI of sensitive genotypes was adversely affected by aridity and salinity. The PI of ICARDA20 and Konouz decreased by approximately 18% and 33%, respectively, when irrigated with non-saline water. The reduction was even greater, reaching 39%, for both genotypes when irrigated with saline water. However, tolerant genotypes Souihli and Batini 100/1B were less impacted by these stress factors.

The fluorescence study provided insights into the photosynthetic apparatus of barley genotypes under stress. It enabled reliable salinity tolerance screening. Furthermore, the study confirmed that the chlorophyll a fluorescence induction curve had an inflection point (step K) even before the onset of visible signs of stress, indicating physiological disturbances, making chlorophyll fluorescence an effective tool for identifying salinity tolerance in barley.

## 1 Introduction

According to the Food and Agriculture Organization (FAO, 2017), the global population is expected to reach 10 billion by 2050. As a result, the demand for food is also expected to increase. To meet each individual’s needs and requirements, the production of cereal crops must double, especially in developing countries. Cereal crops play a vital role in providing food security and social stability in the Mediterranean and the Middle East and North Africa (MENA) regions. However, crop yields in these regions are subject to fluctuations due to factors such as low soil fertility, water and soil salinity, and irregular rainfall. These factors are expected to worsen in the future due to climate change. To overcome the current production limitations, it is essential to find sustainable field methods to improve crop productivity, especially for staple food crops, to ensure food security. Therefore, agricultural research faces a great global challenge in finding ways to enhance crop yields and develop genotypes that can tolerate extreme weather and soil conditions.

Barley (*Hordeum vulgare* L.) is one of the most important cereal crops in the world, ranking just behind maize, wheat, and rice. It is widely used for food, feed, and malt production. Barley is known for its high tolerance to various abiotic stresses such as salinity and drought, making it an ideal plant for stress biology research (Gürel et al., 2016). However, in some countries, like Tunisia, the yields of barley are currently lower than 1092 kg/ha (FAOSTAT, 2022). Therefore, it is essential to phenotype barley genotypes to select suitable and high-yielding cultivars for better barley production. On the other hand, many researchers have promoted the use of new selection and field screening tools for abiotic stress. One of these tools is photosynthetic indices proposed by Kalaji et al. (2017); Stirbet et al. (2018), and Guidi et al. (2019) in the selection process.

Photosynthesis is a fundamental physiological and biochemical process in green plants and organisms that utilizes sunlight to synthesize sugars from carbon dioxide and water, thereby playing a central role in the biomass equation and respiration (Collalti et al., 2020). Chlorophyll is a crucial component for the internal physiological growth and production of oxygen in plants that undergo photosynthesis. Improving and optimizing photosynthesis is considered a vital strategy for enhancing crop yields, as it is a basic process that enables green plants to produce organic material by converting light energy into chemical energy in the form of the nicotinamide adenine dinucleotide phosphate (NADPH) and Adenosine triphosphate (ATP) to synthesize organic compounds. The light phase of photosynthesis comprises three primary subphases. Firstly, the absorption of light and the transfer of excitation energy within the pigment antenna, followed by its trapping at the reaction centers. Secondly, the transport of electrons, which involves the transfer of an excited electron in an oxidation-reduction chlorophyll molecule to an intermediate acceptor, namely, pheophytin in Photosystem II [PSII] or chlorophyll in Photosystem I [PSI]. Thirdly, the stabilization of the energy of electrons during oxidation-reduction, which leads to the photosynthetic transport of electrons, the proton motive force, and the generation of ATP. In turn, this process facilitates the formation of reducing power in the form of NADPH (Kalaji et al., 2017).

Chlorophyll (Chl) fluorescence is a powerful tool for quantifying the different stages of photosynthesis. Kautsky and Hirsch (1931) highlighted the relationship between the initial reactions of photosynthesis and the fluorescence of chlorophyll a (chl-a). The emission intensity of chl-a fluorescence produces a typical curve over time, known as the chlorophyll a fluorescence induction curve.

The OJIP test, proposed by Strasser and Strasser in 1995, is used to measure the function of PSII by translating fluorescence transient measurements into various expressions that quantify its biophysical and phenomenological properties (Tóth et al., 2007). The test starts with the illumination of a dark-adapted plant leaf with continuous light. The fast phase of the fluorescence induction kinetics, which lasts for approximately 1 second, begins to rise rapidly from the origin (O) to maximum fluorescence (Fm), passing through two inflections (J and I) on its way to a peak (P). This process is called the O-J-I-P test (Govindjee, 1995; Strasserf et al., 1995; Papageorgiou et al., 2007; Malapascua et al., 2014).

Stirbet et al. (2018) defined Various Chl fluorescence parameters based on this transient. The induction of chlorophyll fluorescence is a widely used method in photosynthesis research, as it is non-invasive, very sensitive, quick, and easy to measure, and contains important information about the photosynthetic apparatus. However, it requires relatively inexpensive equipment (Lazár, 1999).

Fluorescence varies between an initial level (F_0_) and a maximum level (F_M_) of the curve, called O-J-I-P (Strasser and Govindjee, 1992; Strasserf et al., 1995). The minimal fluorescence, F_0_, is defined as the fluorescence when all the reaction centers of the photosystem II (RCIIs) are open, i.e., when the first quinone electron acceptor of PSII, the first electron acceptor of PSII (QA), is oxidized (Strasser and Stirbet, 2001; Lazar, 2006). Maximal fluorescence F_M_ is defined as the fluorescence when all the RCIIs are closed, i.e., when all QA is reduced (Strasser and Stirbet, 2001; Lazar, 2006). Some of the parameters calculated using the O-J-I-P test are related to energy fluxes for light absorption (ABS), trapping (TR) of excitation energy, and electron transport (ETR) per reaction center (RC) or per sample area called cross-section (CS).

Chl a fluorescence induction (transient) is measured by exposing dark-adapted samples to high light, and it shows a polyphasic rise that has been the subject of extensive research over several decades (Lazár, 1999). The transition O-J represents the reduction of the quinone, the primary quinone electron acceptor QA, which corresponds to the reduction of the acceptor side of Photosystem II (Strasser and Govindjee, 1992; Chowaniec and Rola, 2022).

Chlorophyll fluorescence is a dependable method for detecting stress (Bahrami et al., 2019; Cai et al., 2020). A decrease in fluorescence intensity can be observed after each step, depending on the environmental conditions and stress (Srivastava et al., 1995). Chlorophyll fluorescence has been suggested as a reliable selection criterion in general barley improvement programs (Kalaji and Guo, 2008). Fluorescence parameters have been used as early indicators of salt stress in barley (Jiang et al., 2006; Mehta et al., 2010; Kalaji et al., 2011, 2012; Ahmed et al., 2013) and of heat stress, which is a major constraint on cereal production (Bahrami et al., 2019). The JIP test is used to study the effects of various types of stress, such as heat, drought and salinity.

This research aimed to evaluate the effectiveness of chlorophyll fluorescence in selecting and breeding barley varieties that can survive in dry and saline conditions in the field. We hypothesize that chlorophyll fluorescence can be a valuable technique in distinguishing between different barley genotypes that exhibit varying degrees of tolerance to salinity stress under challenging growth conditions in real field settings.

This paper showcases the findings obtained from experiments carried out in real-world situations that involved challenging environmental conditions, such as salinity and arid climate. These experiments were a part of adaptability trials that were conducted in five distinct biophysical environments. It is important to note that many similar previous studies focused on barley (*Hordeum vulgare* L.) that was grown in growth chambers using vermiculite moistened with half-strength Hoagland solution or in pots for early-stage data collection. These controlled settings are significantly different from real-world conditions, and this study provides valuable insights into the adaptability of barley in challenging environments.

## 2 Materials and methods

### 2.1 Plant material, experimental design and treatments

This study was performed to evaluate the influence of aridity and salinity on PSII of barley (*Hordeum vulgare* L.) under natural field conditions. Three sensitive and three tolerant (Hammami et al., 2017) barley genotypes of different origins were selected from a preliminary screening of 40 genotypes (Hammami et al., 2017), including three salt-tolerant and three salt-sensitive genotypes (**Table 1**), were evaluated during two cropping seasons: 2012–2013 and 2013–2014. Three of the genotypes (100/1B, ICARDA 20, and Barjouj) were obtained from the International Center for Biosaline Agriculture (ICBA), Dubai, United Arab Emirates.

**Table 1:** Categories and origin of the barley genotypes used in the study.

Genotype	Breeding status	Origin	Salt-tolerance	Reference
Konouz	Commercial genotype	Tunisia (North)	sensitive	(Hammami et al., 2016)
Rihane	Commercial genotype	Tunisia	sensitive	(Hammami et al., 2017)
ICARDA 20	Cultivar	ICARDA	sensitive	(Hammami et al., 2016)
Batini 100/1B	Landrace	Oman	tolerant	(Abdullah et al., 2012) (Hammami et al., 2020)
Barjouj	Cultivar	Libya	tolerant	(Hammami et al., 2017)
Souihli	Landrace	Tunisia (Mahdia)	tolerant	(Sbei et al., 2012)

The experiments were conducted in three different locations that represent a gradient of aridity. These locations include Lafreg-Beja (BEJ: 36°44’01.13N; 9°02’14.30E), Barrouta-Kairouan (KAI: 35°34′34.97” N), and El Fje-Medenine (MED: 33°26′54” N, 10°56′31” E). These experiments were located in the humid north, semi-arid central, and southeast arid regions of Tunisia. Tunisia has a Mediterranean climate with very hot, dry summers and cool, wet winters. Rainfall varies greatly from north to south and is not consistent. Bioclimatic zones have been identified in Tunisia by Emberger (1960), based on precipitation. These zones are humid (>800mm) where the BEJ site is installed, sub-humid (600 to 800 mm), semi-arid (400 to 600 mm) where the KAI site is installed, arid (100 to 300 mm) where the MED site is installed, and desert or Saharan (<100 mm). However, rainfall is not the only determining factor as winter temperatures are also important in this context.

**Figure 1:**
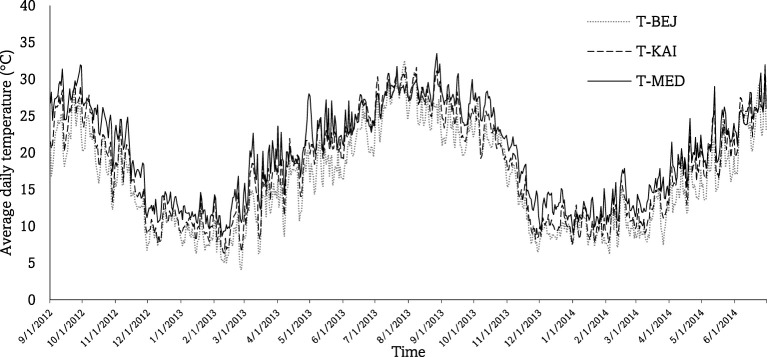
Mean daily air temperature and rainfall of the three experimental sites, Beja (BEJ), Kairouan (KAI), and Medenine (MED), measured on the cropping sessions 2012/13 and 2013/14.

To better characterize environments, precipitation and temperature data were collected from weather stations (Weathereye, Bardon, Leicestershire, Royaume-Uni) installed in each site (**Figure 1**).

The table below presents a summary of the monthly rainfall amounts (in millimeters) and estimated Growing Degree Days (GDD) for the cultivation of barley. The temperature base is set at 5.5°C and the upper limit at 30.0°C. The variation in GDD across the three environments is an effective weather-based indicator that helps to evaluate crop growth. It clearly demonstrates the differences between the three sites and their respective environments (**Table 2**).

**Table 2:** Monthly rainfall (mm) Growing degree days (GDD) of the barley of the three experimental sites: Beja (BEJ) Kairouan (KAI) and Medenine (MED) measured on the cropping sessions 2012/13 and 2013/14.

Site/Month		November	December	January	February	March	April	May	June	Total
Bej 2012/13	Rain	58.2	0.7	139.3	100.2	148.5	64.4	14.1	1.3	526.7
	GDD	312.9	168.9	148.5	100.4	249	323.2	391.2	463.2	2157.3
Bej 2013/14	Rain	74.4	111.8	59.7	47	134.9	36.4	26.3	44.2	534.7
	GDD	240.2	155.9	165.7	170.3	181.6	299.3	389	507.3	2109.3
Kai 2012/13	Rain	19.3	1	42.8	11.9	31.1	66.3	25	2.6	200
	GDD	343.2	176.9	162.2	130.3	302.8	372.4	469.1	510.7	2467.6
Kai 2013/14	Rain	24	10	45.7	46.1	44.1	15.4	22.8	36.1	244.2
	GDD	264.5	158.5	171.9	189.9	210.7	355.3	452.4	546.1	2349.3
Med 2012/13	Rain	14.2	4.3	20.4	9.3	9.1	32	7	0.5	96.8
	GDD	470.6	305	266.4	227.7	374.2	425.2	513.4	552.1	3134.6
Med 2013/14	Rain	28.4	10.6	16	34.4	24.6	12.3	4.4	10.4	141.1
	GDD	399.5	281.4	281.1	268	314.9	414.4	492.7	585.8	3037.8

The two cropping seasons (2012/13 and 2013/14) were characterized by low rainfall at the arid and semi-arid sites and high rainfall at the Subhumid site. The arid site of MED received an annual rainfall of 96.8 mm in the first season and 141.1 mm in the second season. For the semi-arid Site KAI, annual rainfall was of the order of 200 mm and 244.2 mm, respectively, during the first and second season. In the Subhumid BEJ site, annual rainfall was of the order of 530 mm during the two agricultural seasons. Arid and semi-arid climates prevail in the experimental area, with hot, dry to wet summers (May to October) and cool, dry winters (November to April). The maximum temperatures recorded at the two arid and semi-arid sites during the various seasons were 31°C and 33°C, respectively. The minimum temperatures recorded were 5°C and 12°C in KAI and MED, respectively. Temperatures at the BEJ site were lower by two to three degrees.

In Lafreg-Beja, the six genotypes were cultivated under rainfed conditions. However, barley genotypes were grown under two contrasting irrigation water-salinity treatments i.e., 1.2 dS.m^-1^. (S1) versus 13 dS.m^-1^(S2) in KAI and 1.5 dS.m^-1^(S1) versus 13.3 dS.m^-1^(S2) in MED. Saline groundwater and potable water (less than 1.5 dS.m^-1^) were used at both sites. The field trial sites were divided into two sub-plots. Each subplot was irrigated by one water salinity treatment. Three blocks were defined perpendicularly to the sub-plots so that both treatments were observed in each block where the genotypes were completely randomized.

Irrigation was applied based on water demands and weather conditions during the growing seasons. The total water supplied was calculated according to each site’s climatic and soil data to obtain the water barley requirement (440 mm). Throughout the experiment, crop evapotranspiration (ETc) was determined to gauge the average irrigation rate, measured in millimeters per day. The data collected from the weather station installed in the field was utilized to estimate the reference evapotranspiration (ET) and the barely irrigation requirement, employing the Penman-Monteith FAO-56 Method and crop coefficient recommended by FAO. Irrigation was provided from planting to the grain filling stage.

In order to ensure that the water supply was consistent, a drip system was used for irrigation, and each sowing row used a drip irrigation line with 4 Lh-1 emitters, each 33cm. As BEJ is located in the rainfed cereal-growing area of Tunisia, no irrigation was applied. Individual plots constituted by 10 rows of 2 m long, spaced by 0.20 m. Only 4 central rows were used as net plots for measurements to avoid the border effect. Plant growth conditions are described previously (Hammami et al., 2017).

### 2.2 Chlorophyll-a fluorescence transient and JIP-test

The measurements were taken during the precise flowering stage of each genotype for every salt treatment and location to compare their response to stress. The measurement was carried out on the flag leaf, which represents the final emergence of the leaf and signifies the shift from crop growth to grain production. The flag leaf plays a crucial role in the process of photosynthesis, providing the majority of the carbohydrates essential for grain filling, and, therefore, it is the most critical leaf for yield potential (Sanchez-Bragado et al., 2014). Chlorophyll-a fluorescence (OJIP) transients were measured from the middle region of the flag leaf in three plants, which were replicated per genotype, in the morning (between 10 am to 11:30 am). A portable multimode chlorophyll fluorometer (OS5p+, Opti-Sciences, Hudson, NH, USA) was used to perform the measurements. The dark-adapted leaves were exposed to a saturation pulse of white LED light with an intensity of 15000 μmol m^-2^ s^-1^ for a duration of 0.8 seconds over a 4 mm2 leaf area. A total of 15 data points were recorded for every genotype. For dark adaptation, to ensure the complete oxidation of PSII and the electron transport chain prior to a saturating pulse of light. The target leaves were covered with specific leaf clips provided with the instrument. The period of dark adaptation was optimized at 20 minutes.

Various derived Fluorescence parameters and JIP-test parameters were calculated using the multi-mode chlorophyll fluorometer as described by (Schansker et al., 2003; Wang et al., 2011; Kalaji et al., 2012; Beneragama et al., 2014). The total performance index (PI) can provide quantitative information about the vitality of photosynthetic organisms (Tsimilli-Michael and Strasser, 2008; Oukarroum et al., 2009). The detailed definitions and equations of primary and derived JIP test fluorescence parameters are presented in **Table 3**.

**Table 3:** Nomenclature, equations and definition of the OJIP transient and the specific parameters calculated according to the JIP-test.

Raw values	Definition
F_0_= F50 µs	Fluorescence intensity when all PSII RCs are assumed to be open
F_M_=F_P_	Maximum fluorescence intensity at P phase of OJIP when all PSII RCs are assumed to be closed due to saturation illumination
F_J_	Fluorescence intensity at 2 ms at the J phase of OJIP
F_I_	Fluorescence intensity at 30 ms at the I phase of OJIP
F_K_	Fluorescence intensity at 300µs at the K phase of OJIP
**Fluorescence parameter calculated from the raw values**	
Fluorescence parameter	Definition
Fv= F_M_-F_0_	Maximum variable chlorophyll fluorescence
ABS= TR+DI	Rate of light energy (photons) absorbed by PSII antenna
QA	First electron acceptor of PSII
QB	Second acceptor electrons of PSII
TR	Rate of excitation energy (excitons) trapped by the PSII RCs
DI	Rate of energy dissipation in the PSIIs. in the process other than trapping
ET	Electron transport
Fluorescence reports	Definition
Fv/F_M_	Maximum quantum yield of PSII
(dV/dt)o= 4ms-1(F300µs–F0)/(F_M_– F_0_)	The initial slope of the relative variable fluorescence (dVt/dto): Approximate value of the initial slope of the variable fluorescence curve relative to F300µs.
(dVG/dt)0 = 20 ms-1(F100µs-F_0_)/(F_M_ –F_0_)	Approximate value of the initial slope the variable fluorescence curve relative to F100µs
VJ= (F2ms-F_0_)/(FM-F_0_)	The relative variable fluorescence in the step J at 2ms
VI = (F30ms-F0)/(F_M_-F_0_)	The relative variable fluorescence in the step I at 30 ms
Activity performance	Definition
TR/RC	The trapped (maximum) energy flux (leading to QA reduction) per reaction center (RC)
ET/RC	The maximum electron transport flux (further than QA−) per PSII reaction center (RC)
DI/RC	The dissipation energy flux per PSII reaction center (RC)
ABS/RC=[(F_0_/F_J_)/(F_V_/F_M_)]	Light absorption flux (for PSII antenna chlorophylls) per reaction center (RC)
ET_0_/RC=(F_K_/F_0_)/(F_J_/F_0_);	Electron transport flux per reaction center (t=0)
TR0/ABS = 1-F_0_/F_M_ = φP_0_	Maximum quantum yield of primary photochemistry (φPo)
ET_0_/TR_0 =_ 1-VJ = ψ_0_	Electron transport probability (ψo): Efficiency of trapped energy conserved in electron transport beyond QA
ET_0_/ABS= (1-F_0_/F_M_) (1-V_J_) = φE_0_	The quantum yield of electron transport (φEo): Efficiency of energy absorbed and conserved in the electron transport beyond QA-;
(ABS-TR_0_)/ABS = F_0_/F_M_	Efficiency of energy dissipated between capture and absorption.
Performance index (PI)	Definition
$\left[\gamma_{0}/(1-\gamma_{0})\right]={\text{Chl}}_{RC}/{\text{Chl}}_{antenna}$ $RC/ABS=\left[\left(F_{2ms}-F_{50\mu s}\right)/4\times \left(F_{300\mu s}-\right)\right]\times F_{V}/F_{M}$	The expression γ o/(1–γ o) is, therefore the ratio ChlRC/ChlAntenna and estimated by the JIP test as equal to the ratio of reaction centers and the light absorbance flux
$\left[\varphi p_{0}/(1-\varphi p_{0})\right]\;with\;\varphi p_{0}=\frac{F_{V}}{F_{M}}$	The contribution of light reactions to primary photochemistry corresponds to the efficiency by which an absorbed photon will be trapped by PS II reaction centers.
$\left[\psi_{0}/(1-\psi_{0})\right]$ with $\psi_{0}=1-V_{J}=1-\left[\left(F_{J}-F_{o}\right)/F_{M}-F_{o}\right]$	is the fraction of electrons transported beyond QA− per exciton trapped by the open reaction centers (RCs) of PS II. It is the probability that the energy of a trapped exciton is used for electron transport beyond QA. The contribution of thermal reactions (non-photochemical) is derived
$PI_{total\;}\;=\left[\gamma_{0}/(1-\gamma_{0})\right]\times \left[\varphi p_{0}/(1-\varphi p_{0})\right]\times \left[\psi_{0}/(1-\psi_{0})\right]$	The PI total is the product of the performance index and the probability that an electron can move from the reduced intersystem electron acceptors to the PS I end-electron acceptors [(Tsimilli-Michael and Strasser, 2008) (Oukarroum et al., 2009)]: The performance index is one of the chlorophyll fluorescence parameters indicative of plants’ physiological state and vitality

To illustrate the effect of the aridity and salinity on PSII electron transport, the polyphasic Chlorophyll a fluorescence rise curve for the different barley varieties was plotted on a logarithmic scale. A series of specific Chlorophyll a fluorescence parameters were also plotted on Radar plots.

### 2.3 Statistical analysis

The data collected from six different barley genotypes cultivated in five different environments was analyzed using R software (R 4.0.2). Linear models were used to perform an Analysis of Variance (ANOVA) and to determine the mean differences among the genotypes (G), salt treatments (T), and aridity (A), as well as possible interactions between these factors.

Chlorophyll fluorescence parameters (CFP) for a given variety i, in an environment (Site-Aridity) j, in a salinity k, can be expressed as follows:


$${\text{CFP}}_{\text{ijk}}=\text{μ}+G_{i}+E_{j}+S_{k}+E*S_{jk}+G*E_{ij}+{\text{ε}}_{\text{ijk}}$$

Where μ is the mean genotype tolerance observed in the whole experiment, Gi is the mean effect of the genotype i, Ej is the mean effect of the environment j, ExSjk is the effect of the salinity k in the environment j, GxEij is the particular effect of the genotype i in the environment j, and εijk the residue observed for the genotype i in the salinity k of the environment j.

The Turkey HSD method was used to separate the means at a significance level of P<0.05. Finally, the results were presented graphically using Microsoft Excel 2007 software.

## 3 Results

### 3.1 Chl fluorescence induction curve

**Figure 2:**
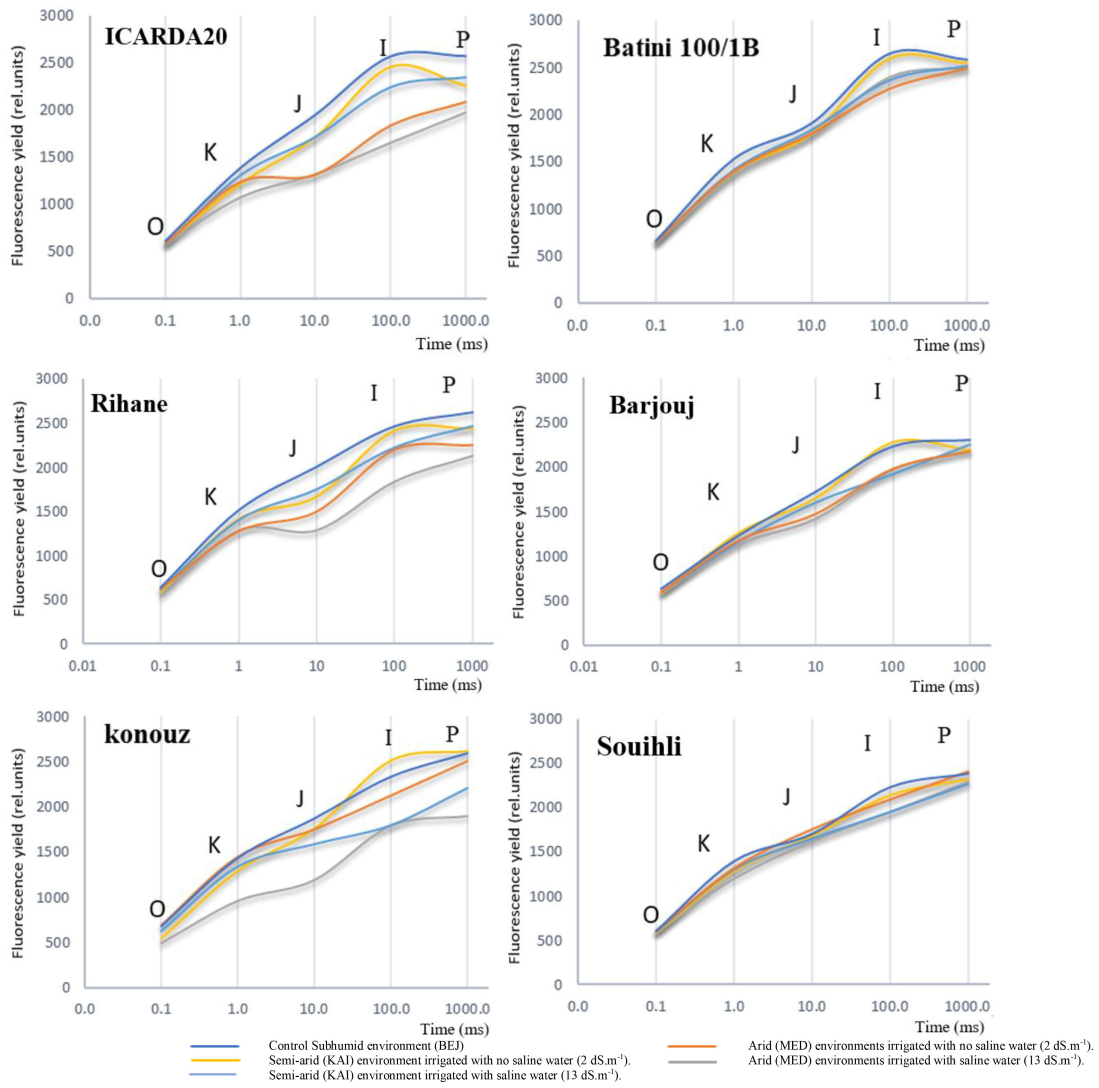
Effect of environmental variation and salt stress on the chlorophyll a fluorescence induction curve in the studied barley genotypes. The curves were plotted on a logarithmic time scale.

In this study, we compared the O-J-I-P curves of different genotypes grown under various environmental conditions. Our results showed that the typical O-J-I-P curve was typical in the humid environment of BEJ site, while the semi-arid site of KAI and arid environment of MED exhibited some stress. Sensitive genotypes displayed a decrease in polyphasic induction typical of fluorescence under salt stress, as seen in **Figure 2**.

We also observed that aridity had no significant effect on the shape of the multiphase O-J-I-P curve of tolerant genotypes such as Batini, Barjouj, and particularly Souihli. However, there was a change in the minimum value of fluorescence (F_0_) for all environments. The parameters F_J_, F_I_, and F_P_ decreased depending on the environment from the humid (BEJ) to the semi-arid site (KAI) to the arid environment (MED), with more significant decreases after increasing the salinity level of irrigation water. Interestingly, changes in the environment did not affect F_J_, F_I_, and F_P_ of the tolerant genotypes Barjouj, Batini, and Souihli, as shown in **Figure 2**.

In all genotypes were observed differences in F_0_ values ranging between 1% and 30% in the arid environments at the MED site when compared to the BEJ site (**Figure 2**). These changes are likely due to the variability in PSII tolerance to high temperatures. Yamane et al. (1997) suggested that minimal fluorescence F_0_ can be an indicator of damage in PSII. However, the increase in the F_0_ level does not necessarily accompany the inactivation of the PS II reaction center (Yamane et al., 1997). The results suggest that this is a reasonable explanation for the Konouz genotype that displayed a 30% decrease in F_0_.

At both KAI and MED sites, the parameters of the Chl fluorescence induction curve F_0_, F_J_, F_I_, and F_P_ decreased by 2.9%, 8.3%, 7.0%, and 5.8% respectively. This decrease was more pronounced after irrigation with saline water (13 dS.m-1), resulting in a decrease of 5.25%, 19.9%, 18.7%, and 12% in F_0_, F_J_, F_I_, and F_P_ parameters, respectively (**Figure 2**).

**Figure 2** shows that salt stress led to a decrease in Maximum fluorescence (F_M_). However, the reduction in F_M_ was less significant in tolerant genotypes compared to susceptible ones. The results of the comparison between the humid environment BEJ, the semi-arid site KAI, and the arid environment MED indicate that the FM reduction was around 2% to 4% for the tolerant genotypes Souihli and Batini (100/1B). In contrast, the reduction in F_M_ was more substantial, ranging between 14% and 24%, for susceptible genotypes ICARDA20 and Konouz (**Figure 2**).

#### 3.1.1 The OJ phase

During the study, it was observed that the parameters F_0_ and F_J_ of the amplitude OJ phase were higher in a humid environment at BEJ and a semi-arid environment at KAI, as compared to the arid environment at MED. Furthermore, it was found that when salt stress was added, the OJ phase of the sensitive genotypes was significantly influenced. This indicated that the combined effect of aridity and salinity was more critical than that of aridity alone, which involves low rainfall and high temperature.

The OJIP fluorescence rise kinetics of the sensitive barley genotype showed a gradual shift towards an OKJIP pattern. The addition of a new intermediate step called “K” was observed at around 300 µS, which led to this change. This alteration was noticeable due to the induction of the K-step.

When exposed to salt treatment, changes were observed in the major steps of the OJIP curve through an analysis of chlorophyll fluorescence transients (**Figure 2**). These changes could help us understand how the photosynthetic process is damaged. Salinity affected the rate of primary photochemistry, leading to an increase in fluorescence at the O-J phase, which was slightly higher in semi-arid (KAI) and arid (MED) environments.

Indeed, it was observed that the inflection point on the curves became more prominent when moving from the subhumid (BEJ site) and semi-arid (KAI site) to the arid (MED) environments for all genotypes, except for Konouz, which showed some difference in F0.

When the sensitive genotypes Konouz, Rihane, and ICARDA20 were grown under stress, a distinct band appeared at 300µs during the fluorescence intensity phase, which is also the inflection point on the O-J-I-P curves (**Figure 2**). The intensity of this band varied among the tested genotypes under stress caused by aridity and salt stress.

The impact of aridity and salinity, along with the genotype effect, was apparent during the electron transfer process in this phase. The FJ point was reduced by about 35% in the sensitive genotypes Konouz and ICARDA20, and only 4% in the tolerant genotype Souihli at the MED site, where salinity was combined with heat stress, as compared to BEJ (**Figure 2**). Results emphasized that higher temperatures of semi-arid (KAI site) to arid (MED) environments damage the oxygen-evolving complex and decrease electron transport and photosynthesis.

#### 3.1.2 The J-I-P transmission

The difference between the BEJ (subhumid), KAI (semi-arid) and MED (arid) sites became more evident from phase J of the polyphasic transition of the chlorophyll-a fluorescence induction curve, with percentages of 2%, 6% and 20% respectively (refer to **Figure 2**). This negative effect was further amplified due to irrigation with saline water. After 20ms, the plastoquinone pool, which is reduced by the activity of PSII, starts to give electrons to the PSI. It was observed that the reduction of fluorescence was extremely significant in the sensitive genotypes, particularly at the arid site (MED) under salt and heat stress. The percentages of reduction were 32.5%, 29.5% and 20.5% in phases J, I and P respectively. For Souihli genotypes, the reduction percentages were in the order of 12%>4.6%>3% (**Figure 2**).

### 3.2 The parameters of chlorophyll fluorescence

Analysis of the variance of chlorophyll fluorescence parameters (dVt/dt_0_, VJ, TR_0_/RC, TR0/ABS (φP_0_), ET_0_/TR_0_ (ψ_0_)) showed a significant effect at the 1% threshold of salinity (S), varieties (V), Aridity (A) as well as for most of the interaction such as salinity x genotypes (**Table 4**).

**Table 4:** Analysis of the variance of chlorophyll fluorescence parameters: maximum quantum yield of primary photochemistry (φPo), Maximum quantum yield of PSII (F_V_/F_M_), the initial slope of the relative variable fluorescence (dVt/dto), The relative variable fluorescence in the step J (VJ) and Trapped energy flux per reaction center (t = 0) (TR_0_/RC) of different genotypes.

Source of Variation		F_V_/F_M_	dVt/dt_0_	VJ	TR_0_/RC	TR_0_/ABS (φP_0_)	ET_0_/TR_0_ (ψ_0_)
Aridity							
	BEJ	0.764a	1.904a	0.753a	1.281b	0.717c	0.246c
	KAI	0.749a	1.722b	0.599b	1.346a	0.730b	0.400b
	MED	0.715b	1.366c	0.541c	1.381b	0.763a	0.458a
Salinity							
	Rainfed	0.764a	1.904a	0.753a	1.281b	0.717c	0.246c
	2 dS.m^-1^	0.743b	1.589b	0.593b	1.493a	0.760a	0.406b
	13 dS.m^-1^	0.722c	1.499c	0.547c	1.234b	0.733b	0.452a
Variety							
	Barjouj	0.735a	1.676a	0.627a	1.330ab	0.743abc	0.372c
	100/1B	0.741a	1.619ab	0.622ab	1.359a	0.745ab	0.377bc
	Suihli	0.751a	1.617ab	0.610abc	1.403a	0.752a	0.389abc
	Konouz	0.736a	1.587ab	0.591c	1.272b	0.731d	0.408a
	Rihane	0.734a	1.575b	0.588c	1.345ab	0.738bcd	0.411a
	ICARDA20	0.73424	1.622 ab	0.602bc	1.272b	0.734cd	0.397ab
**ANOVA**	**DF**						
Aridity (A)	4	0.180***	20.721***	2.719***	0.600***	0.163***	2.719***
Variety (V)	5	0.016 ns	0.457*	0.099***	0.739***	0.023***	0.099***
Salinity (S)	2	0.041***	0.728***	0.194***	6.002***	0.069***	0.194***
V×S	10	0.089**	3.766***	0.155***	0.849**	0.016***	0.155***
A×V	5	0.032*	0.293 ns	0.012 ns	0.070 ns	0.006**	0.012 ns
A×S	6	0.001 ns	1.671***	0.000***	3.433***	0.090***	0.000 ns
A×V×S	5	0.074***	0.522*	0.077ns	0.231**	0.004*	0.077***
Residuals	420	1.374	15.433	0.935	13.364	0.165	0.935

DF, degrees of freedom; ** *, significant difference at 0,1%; **, significant difference at 1%; *, significant difference at 5%; ns, not significant.

Means with the same letter are not significantly different from each other (P.0.05).

In this study, we found that the variation of trapping variance (TR_0_/RC) was significant for genotype by salinity (V*S) interaction (p<0.01) but not for genotype by environment (A*V) interaction. This suggests that the degree of salinity in the irrigation water was the main factor that influenced photon trapping.

We also observed that the interaction Aridity by Salinity (A*S) was significant, indicating that salinity had a different impact on barley performance depending on the degree of aridity in the climate, mainly temperature. All the sources of variation proposed in this study significantly affected the trapped energy flux of PSII ABS (the maximum quantum yield of primary photochemistry, TR_0_/ABS). This means that the absorption part was sensitive to the single and combined effect of both stress, salinity, and climate aridity.

Furthermore, the interaction (A*V*S) was significant for the parameter ET_0_/TR_0_ (the probability that a trapped exciton moves an electron into the electron transport chain further than QA-) (p<0.01). This suggests that the simultaneous presence of salt stress and the degree of aridity of the climate, mainly temperature, affects the electron transport chain. However, the effect depends on the degree of sensitivity of the genotypes tested.

#### 3.2.1 The initial slope of the relative variable fluorescence (dVt/dto)

The impact of salinity on electron transport on the PSII donor side was analyzed by measuring the variable fluorescence concerning step J (VJ) and the slope at the origin of the relative variable fluorescence curve (dVt/dt_0_) (**Table 5**). The results showed that the Konouz sensitive genotype had a decrease in dVt/dt_0_ of around 12% from the BEJ (subhumid) site under rainfed conditions to the KAI (semi-arid) and MED (arid) sites irrigated with low saline water (less than 2 dS.m-1). In contrast, only a 0.6% decrease was observed in the tolerant genotype for Souhli. This reduction was more significant under salt stress, especially in sensitive genotypes, following irrigation with saline water (13 dS.m-1), where the reduction reached 20% and 7%, respectively, in the same genotypes (**Table 5**).

**Table 5:** The values of dVt/dt0, VJ, TR_0_/RC, TR_0_/ABS, ET_0_/TR_0_, and PI in the different sites, environments (Env) and salinity (TRT) in the different barley varieties studied.

SITE	Env	TRT	VAR	dV/dt0	VJ	TR_0_/RC	TR_0_/ABS	ET_0_/TR_0_	PI
BEJ	humid	Rainfed	ICARDA20	1.58 ± 0.17	0.68 ± 0.03	0.58 ± 0.02	0.76 ± 0.03	0.32 ± 0.03	5.26 ± 0.03
KAI	semi-arid	13 dS.m^-1^		1.74 ± 0.17	0.49 ± 0.05	0.89 ± 0.02	0.72 ± 0.04	0.51 ± 0.05	2.26 ± 0.49
KAI	semi-arid	2 dS.m^-1^		1.66 ± 0.17	0.64 ± 0.03	0.64 ± 0.03	0.75 ± 0.03	0.36 ± 0.03	4.17 ± 0.36
MED	arid	13 dS.m^-1^		1.43 ± 0.21	0.53 ± 0.04	0.67 ± 0.124	0.71 ± 0.05	0.47 ± 0.04	2.01 ± 0.48
MED	arid	2 dS.m^-1^		1.51 ± 0.13	0.68 ± 0.05	0.56 ± 0.08	0.74 ± 0.04	0.32 ± 0.05	4.42 ± 0.57
BEJ	humid	Rainfed	Konouz	1.66 ± 0.17	0.67 ± 0.03	0.62 ± 0.03	0.75 ± 0.03	0.33 ± 0.03	4.58 ± 0.42
KAI	semi-arid	13 dS.m^-1^		1.81 ± 0.17	0.56 ± 0.04	0.80 ± 0.01	0.70 ± 0.03	0.44 ± 0.04	1.97 ± 0.41
KAI	semi-arid	2 dS.m^-1^		1.91 ± 0.17	0.66 ± 0.03	0.72 ± 0.03	0.72 ± 0.03	0.34 ± 0.03	3.52 ± 042
MED	arid	13 dS.m^-1^		1.91 ± 0.21	0.52 ± 0.04	0.92 ± 0.04	0.71 ± 0.09	0.48 ± 0.04	1.89 ± 0.41
MED	arid	2 dS.m^-1^		1.51 ± 0.08	0.60 ± 0.03	0.62 ± 0.118	0.72 ± 0.04	0.40 ± 0.04	2.77 ± 0.73
BEJ	humid	Rainfed	Rihane	1.76 ± 0.18	0.69 ± 0.03	0.64 ± 0.01	0.76 ± 0.04	0.31 ± 0.03	5.25 ± 0.74
KAI	semi-arid	13 dS.m^-1^		1.60 ± 0.09	0.54 ± 0.03	0.74 ± 0.07	0.72 ± 0.05	0.46 ± 0.03	2.15 ± 0.36
KAI	semi-arid	2 dS.m^-1^		1.70 ± 0.18	0.61 ± 0.03	0.69 ± 0.01	0.75 ± 0.04	0.39 ± 0.02	3.57 ± 0.67
MED	arid	13 dS.m^-1^		1.88 ± 0.19	0.47 ± 0.04	1.00 ± 0.01	0.75 ± 0.04	0.53 ± 0.03	2.02 ± 0.42
MED	arid	2 dS.m^-1^		1.79 ± 0.04	0.58 ± 0.04	0.76 ± 0.04	0.76 ± 0.03	0.42 ± 0.03	3.34 ± 0.62
BEJ	humid	Rainfed	Souihli	1.78 ± 0.14	0.62 ± 0.03	0.72 ± 0.01	0.74 ± 0.04	0.38 ± 0.03	3.46 ± 0.41
KAI	semi-arid	13 dS.m^-1^		1.69 ± 0.15	0.63 ± 0.04	0.67 ± 0.01	0.74 ± 0.03	0.37 ± 0.04	3.55 ± 0.37
KAI	semi-arid	2 dS.m^-1^		1.58 ± 0.14	0.64 ± 0.03	0.62 ± 0.01	0.75 ± 0.05	0.36 ± 0.03	3.86 ± 0.66
MED	arid	13 dS.m^-1^		1.42 ± 0.08	0.60 ± 0.06	0.59 ± 0.08	0.74 ± 0.04	0.40 ± 0.06	3.23 ± 0.42
MED	arid	2 dS.m^-1^		1.61 ± 0.08	0.63 ± 0.05	0.64 ± 0.04	0.75 ± 0.09	0.37 ± 0.05	3.79 ± 0.41
BEJ	humid	Rainfed	Barjouj	1.43 ± 0.1	0.65 ± 0.08	0.55 ± 0.02	0.72 ± 0.03	0.35 ± 0.08	3.52 ± 0.16
KAI	semi-arid	13 dS.m^-1^		1.47 ± 0.12	0.56 ± 0.04	0.66 ± 0.02	0.72 ± 0.05	0.44 ± 0.08	2.37 ± 0.61
KAI	semi-arid	2 dS.m^-1^		1.36 ± 0.21	0.61 ± 0.04	0.56 ± 0.02	0.73 ± 0.04	0.39 ± 0.04	3.11 ± 0.44
MED	arid	13 dS.m^-1^		1.36 ± 0.14	0.52 ± 0.06	0.65 ± 0.08	0.73 ± 0.05	0.48 ± 0.04	2.21 ± 0.45
MED	arid	2 dS.m^-1^		1.68 ± 0.21	0.66 ± 0.04	0.63 ± 0.06	0.73 ± 0.06	0.34 ± 0.06	3.94 ± 0.43
BEJ	humid	Pluvial	Batini (100/1B)	1.80 ± 0.24	0.65 ± 0.04	0.70 ± 0.02	0.74 ± 0.06	0.35 ± 0.04	3.98 ± 0.42
KAI	semi-arid	13 dS.m^-1^		1.62 ± 0.32	0.62 ± 0.07	0.65 ± 0.01	0.74 ± 0.06	0.38 ± 0.07	3.56 ± 0.54
KAI	semi-arid	2 dS.m^-1^		1.64 ± 0.29	0.64 ± 0.04	0.64 ± 0.02	0.74 ± 0.08	0.36 ± 0.04	3.69 ± 0.37
MED	arid	13 dS.m^-1^		1.52 ± 0.24	0.61 ± 0.03	0.62 ± 0.04	0.74 ± 0.03	0.39 ± 0.03	3.30 ± 0.37
MED	arid	2 dS.m^-1^		1.55 ± 019	0.60 ± 0.05	0.64 ± 0.04	0.74 ± 0.03	0.40 ± 0.05	3.24 ± 0.435

Moreover, the decrease in fluorescence variable concerning stage J (V_J_) was 27.9% and 22% for the sensitive genotype ICARDA20, at KAI and MED compared to BEJ. A reduction was also observed in Konouz, which was 16.4% and 22.3% in the same order. However, this reduction did not exceed 2% in both environments for tolerant genotype Souihli (**Table 5**).

#### 3.2.2 The maximum quantum yield of primary photochemistry TR_0_/ABS (φPo)

The flow ratio of the absorbed energy fraction (TR_0_/ABS), which is an indicator of the maximum quantum yield of primary photochemistry in PSII (φP_0_), remained stable for most tolerant genotypes (0.74) (**Table 4**). However, Konouz and ICARDA20 showed a slight decrease in φP_0_. Without salt stress, φP_0_ ranged between 0.75 and 0.76 at BEJ, and between 0.74 and 0.75 at KAI and MED. Under salt stress, it varied between 0.71 and 0.72. The exposure of barley to aridity (high temperature) and salinity stress led to a decrease in φP_0_.

#### 3.2.3 The capture of the photon (exciton) by the reaction center “Trapping” TR_0_/RC

The susceptibility of certain genotypes to aridity has led to an increase in TR_0_/RC. In particular, ICARDA20 and Konouz experienced a 10% and 13.8% increase respectively (**Table 5**). When exposed to both salt stress and aridity, the same genotypes experienced a 34% and 29% increase respectively (**Table 5**). The tolerant genotypes Batini 100/1B and Souihli did not experience a significant increase in the simple effect of aridity nor under the combined stress of aridity and salinity.

### 3.3 Performance index

The performance index (PI) of sensitive genotypes was adversely affected by aridity and salinity, as shown in **Table 5**. The PI of ICARDA20 and Konouz decreased by approximately 18% and 33%, respectively, when irrigated with non-saline water. The reduction was even greater, reaching 39%, for both genotypes when irrigated with saline water. However, tolerant genotypes were less impacted by these stress factors. It is noteworthy that the most significant decrease in performance was observed in the sensitive genotypes under salinity stress.

### 3.4 Presentation of the parameters of the effect of salt stress and arid climate in the form of a radar graph

The effect of stress on the photosynthetic apparatus was observed on the following parameters [(Kalaji et al., 2011; Wang et al., 2011; Beneragama et al., 2014)]:

- V_J_ = (F_J_-F_0_)/(F_M_-F_0_) represents the relative variable fluorescence at time J.
- V_I_ = (F_I_-F_0_)/(F_M_-F_0_) represents the variable fluorescence at time I (30 ms).
- φo/[1-φo] a ‘conformation’ term for primary photochemistry: represents the performance of the photochemical reactions.
- Ψo/[1-ψo] a ‘conformation’ term for thermal reactions (nonlight dependent reactions): represents the performance of non-photochemical reactions.
- RC/ABS density of reaction centers per PSII antenna chlorophyll.
- ET0/RC, maximum electron transport flux (further than QA−) per PSII reaction center (RC).
- RC/CSM, number of active reaction centers per section of the illuminated leaf.
- ABS/CSM, absorption by a selection of illuminated leaf.
- TR0/CSM, capture energy per section of the illuminated leaf.

**Figure 3:**
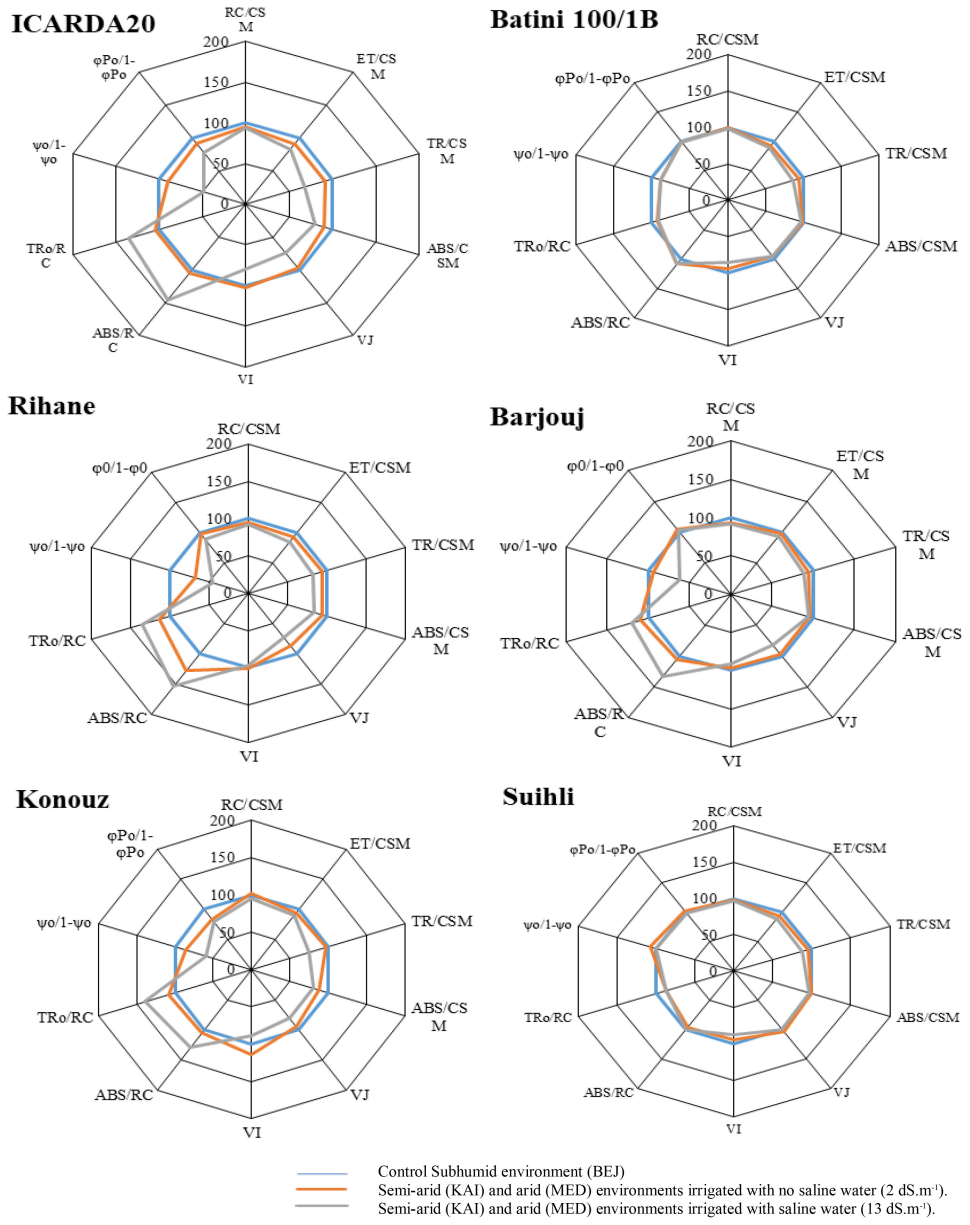
Effect of the climate aridity and salt stress on selected parameters characterizing the performance of Photosystem II of the six studied barley genotypes. The curves were plotted on a logarithmic time scale.

**Figure 3** shows all the parameters described in a radar chart of the three tolerant and salinity-sensitive varieties. The Subhumid environment with the absence of stress is considered as a control (blue line).

Each parameter is represented as values of the KAI and MED site irrigated by low saline water (orange line) or saline water (grey line) compared to the control (blue line). This representation highlights the effect of the arid climate (orange line) and the combined aridity/salinity effect (grey line).

The study showed that all genotypes experienced a decline in the RC/CSM, ABS/CSM, TR_0_/CSM parameters and the VJ and VI parameters due to the arid climate and salt stress. Interestingly, the three sensitive genotypes, Konouz, ICARDA20, and Rihane, were affected the most. **Figure 3** provides a visual representation of these findings. The decrease in the TR/CSM parameter indicated a reduction in the re-oxidation of QA-. The ET_0_/CSM parameter decreased at the KAI and MED sites, meaning that the arid climate prevented the transport of electrons by the reaction center. In addition, irrigation with saltwater reduced electron transfer, likely due to the denaturation of native protein that provides some solvent shielding to the quinone. The ABS/CSM parameter, which is proportional to chlorophyll concentration, demonstrated that the amount of chlorophyll synthesized in the tolerant genotypes was not affected by abiotic stresses such as salinity and aridity of the climate.

The TR_0_/RC and ET0/ABS parameters showed a steady increase in three sensitive Konouz genotypes. ICARDA20 and Rihane genotypes were found to be mainly affected by salt stress, as shown in **Figure 3**. The increase was more pronounced when the plants were irrigated with saline water, reaching up to 30%. Salinity leads to an increase in the effective antenna size per reaction center (ABS/RC), particularly in ICARDA20 and Konouz genotypes, especially when combined with aridity stress.

## 4 Discussion

### 4.1 Chl fluorescence induction curve

The O-J-I-P multiphase transients exhibit variable curves depending on different environmental conditions and the salinity level of irrigation water. This variability makes it possible to analyze the function of the photosynthetic apparatus and use it as a trait to study the site adaptability of barley. In the current study, the trends of the Chl fluorescence induction curve were also a function of different genotypes. The tolerant varieties, Souihli, Barjouj, and 100/1B showed less remarkable differences between the induction curve’s characteristic points under different stresses than the susceptible varieties, Konouz, ICARDA20, and Rihane, which exhibited a significant response.

The observed decrease in F_M_ of chlorophyll fluorescence of the tested sensitive genotypes was probably due to the denaturation of the PSII reaction center proteins (Weng and Lai, 2005) following a risk of denaturation of chlorophyll-proteins in response to aridity, primarily high temperature (Yamane et al., 1997) and salinity (Jiang et al., 2006; Akhter et al., 2021). Furthermore, Bahrami et al. (2019) indicated that grain yield loss in barley under heat stress was inversely correlated with the maximum quantum yield of PSII photochemistry (Fv/Fm) and chlorophyll content.

Measuring chlorophyll fluorescence and its effect on PSII electron transport can help determine the impact of salinity on barley. Chowaniec and Rola found this to be an effective approach in their study in 2010. Similarly, Kalaji et al. discovered that the “Arabi Aswad” genotype of barley was more tolerant to salinity than the “Arabi Abyad” genotype using a similar approach. These findings support previous observations that the initial photosynthetic reactions of barley play a significant role in salt stress tolerance, as noted by Zeeshan et al., 2020. Other crops, such as wheat (Betzen et al., 2019) and tomato (Kamanga et al., 2020), also experienced a reduction in total chlorophyll content due to salinity.

The OJ phase of the chlorophyll fluorescence curve of the sensitive genotypes was significantly impacted by the combined effect of arid climate and salinity, more so than by the sole effect of this phase. The O-to-J rise indicates the photochemical phase, reflecting primary photochemistry, i.e., the reduction of QA. According to Lazar in 1999, environmental factors such as temperature and salinity can affect this phase. A partially reversible decrease in the quantum yield of PS II photochemistry caused a slight variation in F_0_, while an irreversible disconnection of the small light-harvesting complex of PS II caused a higher variation in F_0_, as explained by Lazar in the same study.

During the 300µs phase of the OJIP curves, a band appears in the fluorescence intensity, known as the K band. This band varied between the genotypes under saline and/or thermal stress in the arid site. However, for the Souihli genotype, the K point at phase 300µs remained unaffected by either the arid climate or salinity, indicating its adaptation to arid areas with high salinity.

The study showed that specific parts of the OJIP curve are affected by different types of plant stress, and only the sensitive genotypes showed a K step at 300 µs. Thus, chlorophyll fluorescence was confirmed as an efficient selection criterion for salinity and climate aridity tolerance in barley genotypes. The appearance of the K band coincides with electron donor limitations, as observed in previous research.

An analysis of chlorophyll fluorescence transients revealed some changes in the major steps of the OJIP curve when subjected to salt treatment (**Figure 2**). Indeed, the sensitive barley genotype’s fluorescence rise kinetic OJIP gradually transitioned into an OKJIP pattern, with the addition of a new intermediate step labeled “K” that occurs at roughly 300 µS. This change was accompanied by the induction of the K-step. Similar patterns have been observed for other species, such as moss (Ren et al., 2023).

These modifications could help in understanding the mechanism of damage to the photosynthetic process. In particular, salinity affected the rate of primary photochemistry, resulting in an increase in fluorescence at the O-J phase, which was slightly higher in semi-arid (KAI site) and arid (MED) environments. The appearance of a small K-band (**Figure 2**) indicated a disturbance during the water decomposition at the OEC. Trotti et al. (2023) confirmed this finding.

The water-splitting system of the donor side of PSII might be seriously impacted by water salinity, leading to inactivation of the oxygen-evolving complex (OEC) center; this could be confirmed by the positive K-band of the O-J phase. The damage to the OEC (inactivation of RCs) prevented the overproduction of reduced QA^−^, which might be a plant response to avoid photoinhibition.

Damage to the OEC is the primary step of the so-called two-step hypothesis to reveal the molecular mechanism of the primary photodamaging reaction (Nishiyama et al., 2006; Schreiber et al., 2012), and the secondary damage occurred in the RC of PSII, leading to an increased lifetime of P680+ and the formation of singlet oxygen (Schreiber et al., 2012). The level of salinity in irrigation water may harm the water-splitting system in the donor side of PSII of the sensitive genotypes Konouz, Rihane, and ICARDA20. It may cause inactivation of the oxygen-evolving complex (OEC) center, which is confirmed by the positive K-band of the O-J phase.

When the OEC is damaged, it hinders the overproduction of reduced QA^−^, which is a plant response to avoid photoinhibition. It is the primary step in the two-step hypothesis, which explains the molecular mechanism of the primary photodamaging reaction (Nishiyama et al., 2006; Schreiber et al., 2012; Zhao et al., 2017). Secondary damage occurs in the RC of PSII, leading to an increased lifetime of Photosystem II primary donor (P680+) and the formation of singlet oxygen (Schreiber et al., 2012; Zhao et al., 2017). The inefficient reduction of P680+ resulted in a decrease in charge separation efficiency, as indicated by the Fv/Fm (Zhao et al., 2017).

The observed impact on the sensitive genotypes in the presence of high salinity is also possible because that the ability to synthesize proteins is reduced, which can affect the synthesis of the water-oxidizing enzyme system (Roşca et al., 2023). This, in turn, can alter the entire process of photosynthetic electron transport between photosystems, leading to limited re-oxidation of the PQ pool (Zhao et al., 2017). Low availability of terminal electron acceptors can lead to a mostly reduced PQ pool under salinity and aridity conditions, which in turn can cause acceptor-side PSII photoinhibition and the formation of ROS. Similar results were observed with the impact of glyphosate; Tao et al. (2022) observed the presence of a K-band, which is attributed to an imbalance in the electron transfer from the PSII donor side to the acceptor side and can be used to indicate the activity of OEC.

In addition to salinity, the present study investigated the impact of environment or aridity indicated by high temperatures on the visibility of the K point in semi-arid (KAI site) to arid (MED) environments. The results revealed a significant increase in the visibility of the K point for the sensitive genotypes Konouz, Rihane, and ICARDA20 at higher temperatures, indicating damage to the oxygen-evolving complex (OEC) due to the impact of high temperatures. In contrast, the tolerant genotypes exhibited a slight variation in the K point (**Figure 2**). The research findings underscore that higher temperatures in semi-arid (KAI site) to arid (MED) (**Figure 2**) environments inflict damage to the oxygen-evolving complex, leading to a decrease in electron transport and photosynthesis. Similar observations were made by Mathur et al. (2023) regarding soybean, where higher temperatures (40/32°C) were found to have adverse impacts.

These results provide a better understanding of the impact of high temperatures on plant growth and can be used to inform future research in this field.

The study reinforces the results observed in the work of Srivastava et al. (1997), who reported that stage K occurs naturally in endemic plant species in arid and warm environments even before the onset of visual signs of stress. Under certain conditions, additional steps, such as the K, G, and H steps, can appear in the chlorophyll a fluorescence induction curve. Therefore, step K can be an indicator of physiological disturbances. However, major variations occurred with Konouz and ICARDA20 genotypes.

The 2ms stage J provides information on the events of photochemistry’s primary reactions, mainly the reduction of QA. Genotypes Rihane, Konouz and ICARDA20 had variations during the J phase, reflecting the exchange of a plastoquinol molecule PQH2 with a plastoquinone molecule at the QB site. As a result, step J represents an accumulation of the QA-QB form (Srivastava et al., 1995).

The reduction of fluorescence in the J-I-P transient was particularly noticeable in sensitive genotypes, especially at the arid site (MED) under salt and heat stress. Differences among the study sites became more evident from phase J of the polyphasic transition of the chlorophyll a fluorescence induction curve, with negative impacts becoming more apparent after irrigation with saline water. According to a study by Lazar, 2006, J-I-P transient cannot be speeded up by further increasing the intensity of exciting light and it was called the thermal phase of the chlorophyll a fluorescence induction curve because it depends on the temperature of measurement (within physiological range). The current study confirmed this phenomenon under different environments (temperature and salinity), where significant variation was observed, mainly for the sensitive genotypes.

The JI phase may reflect the partial reduction of the plastoquinone pool. Step I (at ~ 30ms) can express the reoxidation of a plastoquinone molecule by the cytochrome complex (Cyt b6f). During the IP phase of the OJIP fluorescence curve, QA continues to decrease to QA- until it is wholly reduced, i.e., after 20ms (**Figure 2**) (Strasser and Stirbet, 2001). The tolerant genotype Souihli was the least affected. After 20ms, the plastoquinone pool, which is reduced by the activity of PSII, seems to start giving electrons to the PSI.

A decreased FM was observed for all of the sensitive genotypes Konouz, Rihane, and ICARDA20 after irrigation with saline water, which was slightly higher in the semi-arid (KAI site) and arid (MED) environments. The decrease in FM was similar to observations made in different studies (Canora et al., 2022). Salinity stress leads to a decrease in chlorophyll fluorescence signal, which flattens OJIP curves (**Figure 2**). This phenomenon has been observed in different species exposed to salinity stress. It is attributed to a limitation in the quinones’ reoxidation rate, resulting in a reduced ability of PS II to transfer absorbed light energy to the photosynthetic linear electron transport chain (Canora et al., 2022).

Thus, the chlorophyll a fluorescence induction curve varied under environmental stress effects and the degree of sensitivity of a genotype to these stresses. Consequently, the study of fluorescence was an informative tool to study the various barley genotypes’ varietal responses to these environmental stresses (Allakhverdiev and Murata, 2004), especially salt stress (Kalaji et al., 2011; Wang et al., 2011; Akhter et al., 2021).

The results show that climate aridity impacts plant physiology represented by the parameters of the Chl fluorescence induction curve. The American Meteorological Society defines aridity as the extent to which a climate lacks adequate moisture, which is essential for supporting life. It is the opposite of humidity, and this comparison between aridity and humidity is also known as precipitation effectiveness (AMS 2012).

### 4.2 Chlorophyll fluorescence parameters

The effect of the genotype-salinity interaction on the trapped energy flux per reaction center (TR_0_/RC) was found to be significant. However, the genotype by environment interaction did not have a significant impact. This suggests that the degree of salinity in irrigation water (S) was the main factor that affected photon trapping. Moreover, salinity had varying effects on trapping depending on the degree of aridity, which was mainly driven by temperature.

The parameter (TR/RC) refers to the specific trapping flux at time zero. At any given time, TR/RC represented the rate at which an exciton was trapped by the RC, resulting in the reduction of QA to QA- (Ulfat et al., 2020). This fundamental step in energy flow during photosynthesis was found to be highly sensitive to salinity, as confirmed by the research of Mehta et al. (2010) and Akhter et al. (2021).

Salt stress and the degree of climate aridity significantly impacted specific energy flows. This may also be due to PSII’s reduced capacity to transport electrons under salinity stress (Akhter et al., 2021). Additionally, Ahammed et al. (2018) confirmed the direct relationship between chlorophyll content and the electron transport chain in photosynthesis.

In conclusion, photosynthesis activities are a reliable tool for identifying stress tolerance in barley (Cai et al., 2020). Studies on other crops, such as mustard (Wani et al., 2019) and canola (Ulfat et al., 2020), have observed and confirmed this phenomenon.

### 4.3 The slope of variable chlorophyll fluorescence (dVt/dt_0_)

The effects of salinity on electron transport on the PSII donor side were examined by determining the variable fluorescence concerning step J (VJ) and the slope at the origin of the relative variable fluorescence curve (dVt/dt_0_). This measures the difference between the maximum QA reduction and QA-re-oxidation rates (Srivastava et al., 1995). Results showed that the sensitive genotype experienced a decrease in dVt/dt_0_, especially in sites irrigated with low saline water such as the rainfall subhumid, semi-arid and arid sites. The extent of the reduction varied according to the levels of aridity and was more pronounced under salt stress, particularly in susceptible genotypes, following irrigation with saline water (13 dS.m^-1^). In contrast, an increase of dVt/dt_0_ could be due to an inhibition of the rate of QA-re-oxidation, according to Oukarroum (Oukarroum et al., 2006). Similarly, a decrease in fluorescence variable concerning stage FJ (VJ) was observed, reflecting incomplete re-oxidation of the PQ-pool, as reported by Strasser and Stirbet (2001).

### 4.4 The maximum quantum yield of primary photochemistry TR_0_/ABS (ΦP_0_)

The absorbed energy fraction’s flow ratio (TR0/ABS) indicates the maximum quantum yield of PSII primary photochemistry, also known as φP0. This ratio has been found to be stable for most tolerant genotypes. However, a slight decrease in φP0 has been observed in Konouz and ICARDA20. When exposed to an arid climate, high temperature, and particularly salinity stress, barley has been known to exhibit a decrease in φP0.

### 4.5 The capture of the photon (exciton) by the reaction center “Trapping” TR_0_/RC

An increase in the TR_0_/RC ratio was observed mainly in susceptible plant genotypes under combined salt and aridity stress. For sensitive genotypes, this increase in ratio only occurred when both stresses were present. The TR_0_/RC ratio represents the maximum rate at which a photon is trapped by the RC, leading to reduced QA. An increase in this ratio means that QA was reduced, but their re-oxidation was inhibited, and QA could not efficiently transfer electrons to QB.

The maximum value of TR0/RC is calculated using the JIP-test, which corresponds to the expression of the band K (curve O-J-I-P). This band’s appearance was observed in the OJIP fluorescence induction curve under salinity and aridity stress, as confirmed by Kalaji et al. (2011). This band has also been observed under the influence of water stress (De Ronde et al., 2004). This can be explained by an imbalance between the flow of electrons leaving the reaction centers on the acceptor side and the flow of electrons entering the reaction centers on the donor side (Strasser, 1997). Our results confirm the appearance of this band under salt stress alone or combined with heat stress due to the aridity of the environment. Chlorophyll fluorescence is an effective tool for identifying salinity tolerance in barley, as it can help detect an inflection point (step K) indicating physiological disturbances, even before the onset of visible signs of stress.

### 4.6 Performance index

The impact of aridity and salinity stress factors on six different barley genotypes were studied. It was observed that tolerant genotypes were significantly less affected by these stress factors compared to sensitive genotypes. The performance index (PI) of the photosystem II (PSII) was found to be the most distinguishing parameter among the genotypes tested. Sensitive genotypes showed a reduced PI under both aridity and salinity stress, while tolerant genotypes showed better performance indices. PI is a complex parameter that describes the ratio of reaction center per absorption flow, the maximum quantum yield for primary photochemistry, and the quantum yield for electron transport. Therefore, it can be used to differentiate plants’ responses to stress, especially for salt stress responses (Kalaji et al., 2011; Küpper et al., 2019).

### 4.7 Effect of salt stress and arid climatic environments

All genotypes experience a decline in the RC/CSM, ABS/CSM, TR0/CSM, VJ, and VI parameters due to the stress caused by arid climates and, more expectedly, under the effect of salinity. However, this effect is more pronounced in the three sensitive genotypes under salt stress, as depicted in **Figure 3**.

The parameter ABS/CSM, which is proportional to the chlorophyll concentration, showed that the amount of chlorophyll synthesized in tolerant genotypes was not affected by abiotic stresses such as salinity and aridity of the climate.

The TR_0_/RC and ET_0_/ABS parameters increased steadily in the three sensitive Konouz, ICARDA20, and Rihane genotypes, mainly under salt stress. TR_0_/RC represents the maximum rate by which the RC traps a photon by allowing the QA reduction. An increase in this ratio indicates that the QA has been reduced but cannot be re-oxidized due to the thermal stress characteristic of arid climates.

Salinity causes an increase in the effective antenna size per reaction center (ABS/RC), mainly in ICARDA20 and Konouz genotypes. Additionally, a more significant increase in the size of the photosynthetic antenna is essential under higher stress (a combination of salt stress and aridity stress), which is confirmed by (Mehta et al., 2019).

Therefore, ABS/RC demonstrates the average antenna size that expresses the total absorption by the PSII reaction center (RC) collector antennas divided by the number of active reaction centers (in the sense of QA reduction). Nevertheless, since inactive centers have also been added to the total antenna size, the ABS/RC ratio appears to be higher (Luo et al., 2016).

The reduction in the performance of photochemical reactions (φo/[1-φo]) and the performance of no-photochemical reactions (Ψо/[1-Ψо]) due to salinity has been observed in sensitive barley genotypes. According to Demmig-Adams et al. (2005), this reduction resembles the changes occurring under photoinhibition.

The decrease in performance between energy capture and electron transport (Ψо/[1-Ψо]) and between absorption and energy capture (φo/[1- φo]) causes a decrease in photosynthetic performance of genotypes sensitive to abiotic stress (aridity and salinity). Kalaji et al. (2011) have suggested that the sensitive barley genotypes’ susceptibility to salt stress may be, among other things, a result of the low capacity of some pathways such as the Mehler reaction (water-water cycle), which has a protective function in the case of a limited supply of PSII in water. This also helps maintain ATP formation and allows efficient water circulation in the cell in plants with osmotic stress (Osmond and Grace, 1995; Arif et al., 2020).

## 5 Conclusions

The present study aimed to evaluate the photosynthetic activity of six different barley genotypes grown in three locations with varying levels of aridity and salinity. The results showed that the barley genotypes with higher photosynthetic rate and PS II efficiency exhibited better salt tolerance. Fluorescence measurements indicated a decline in activity that was linearly related to an increase in aridity, suggesting that fluorescence can be effectively used to measure the barley genotype responses to salinity and aridity. The individual genotypes were also differentiated based on this method. The OJIP curve analysis revealed that the barley genotypes significantly followed different patterns based on their salinity and aridity tolerance. For the sensitive genotypes (Konouz, Rihane and ICARDA 20), the JIP fluorescence was significantly reduced, and the OJ phase and the K point on the curve were also altered, reflecting growth disturbance due to stress. However, tolerant genotypes such as Souihli exhibited no changes at the K band, which confirmed their tolerance to salinity stress.

The results indicate that the aridity levels and salinity at each site did not affect the amount of chlorophyll-a synthesized by tolerant genotypes. On the other hand, a decrease in energy and electron transport was observed in sensitive genotypes due to their reduced photosynthetic performance under abiotic stress. In addition, the photochemical reactions (φ0/[1-φ0]) in sensitive barley genotypes were related to photo-inhibition.

The use of chlorophyll fluorescence effectively distinguished the performance of aridity and salinity sensitive barley genotypes in marginal environments. This screening through field experiments is a crucial step in the phenotyping of crop genotypes and breeding work in marginal environments. Further field experiments are necessary to validate the utility of this tool across various crops and environments.

## Data availability statement

The raw data supporting the conclusions of this article will be made available by the authors, without undue reservation.

## Author contributions

ZH: Data curation, Formal analysis, Methodology, Writing – original draft, Writing – review & editing. ST-H: Data curation, Writing – review & editing. NN: Writing – review & editing. SR: Data curation, Funding acquisition, Methodology, Project administration, Writing – review & editing. YT: Funding acquisition, Project administration, Supervision, Writing – review & editing.
